# Radiation dose and gene expression analysis of wild boar 10 years after the Fukushima Daiichi Nuclear Plant accident

**DOI:** 10.1038/s41598-022-21436-5

**Published:** 2022-11-04

**Authors:** Motoko Morimoto, Jin Kobayashi, Yasushi Kino

**Affiliations:** 1 School of Food Industrial Sciences, Department of Food Resource Development, Miyagi University, 2-2-1 Hatatate, Taihaku-Ku, Sendai, Miyagi 982-0215 Japan; 2 Department of Chemistry, Tohoku University, Sendai, Miyagi Japan

**Keywords:** Immunology, Ecology

## Abstract

The Fukushima Daiichi Nuclear Power Plant accident led to contamination with radioactive cesium in an extensive environment in Japan in 2011. We evaluated the concentration of radioactive cesium in the skeletal muscles of 22 wild boars and the expression of *IFN-γ*, *TLR3*, and *CyclinG1* in the small intestine and compared them with those of wild boar samples collected from Hyogo prefecture. The average ^137^Cs radioactivity concentration in wild boars in the ex-evacuation zone was 470 Bq/kg. Most of samples still showed radioactivity concentration that exceeded the regulatory limit for foods, but the dose remarkably decreased compared with samples just after the accident. *IFN-γ* expression was significantly higher in wild boars in the ex-evacuation zone than in samples from Hyogo prefecture. *TLR3* expression was also upregulated. *CyclinG1* expression also tended to be high. Hence, wild boars might have received some effects of low-dose radiation, and immune cells were activated to some extent. However, pathological examination revealed no inflammatory cell infiltration or pathological damage in the small intestine of wild boars in the ex-evacuation area. Long-term monitoring would be necessary, but we consider that the living body responds appropriately to a stimulus from a contaminated environment.

## Introduction

On March 11, 2011, the Great East Japan Earthquake was one of the most significant disasters caused by earthquakes and tsunamis. Moreover, the accident at the Fukushima Daiichi Nuclear Power Plant resulted in widespread contamination of radioactive materials. After the accident, more than 165,000 people were evacuated, but wild and livestock animals were left behind in the evacuation zone at that time. We had earlier investigated the effect of radiation on those animals, and the results were published in several research papers^1–6^ and a book chapter^7^. However, because the half-life of ^137^Cesium is approximately 30 years, a long-term environmental survey in the ex-evacuation area is necessary to understand the impact of chronic low-dose radiation on wildlife physiology.

Ten years have elapsed since the earthquake, much of the area where people lived has been decontaminated already, and humans are returning now. Although several people are evacuating, the remaining wild animals are free to live contaminated with radioactive materials. Recent research has revealed that numerous wildlife species are now abundant throughout the ex-evacuation zone^8^. Hunters in Fukushima have exterminated numerous wild animals, but they are not used for human consumption due to the contamination. Even after the Chernobyl accident, wildlife surveys have reported high radioactive contamination rates in wild boars even after several years^9^. In a previous research that examined 213 wild boar muscles in Tomioka town, Fukushima Prefecture, in 2019, it was observed that 98.6% of the samples had radioactivity concentration that exceeded the standard value (100 Bq/kg)^10^ as a general food. Therefore, the meats of those wild boars are not edible and are discarded. However, these wild boars are considered to be affected by low doses of radiation, and analyzing them is important considering the effects on humans.

**Figure 1:**
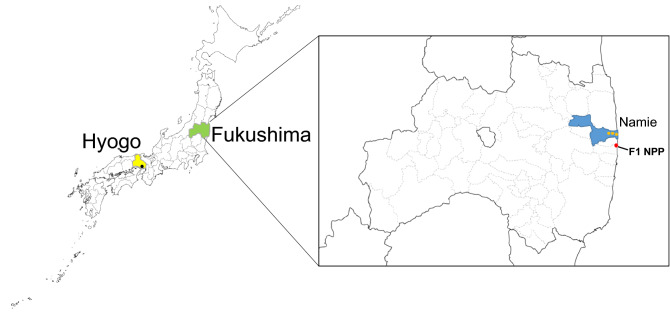
Map of Japan showing the location of Hyogo (yellow) and Fukushima (green) Prefectures where the samples were collected. Orange circles show the sampling site in Fukushima. The black circle indicates the sampling site in Hyogo. The red circle indicates the place of the Fukushima Daiichi Nuclear Power Plant.

The physiological functions and immune systems of pigs are extremely similar to those of humans^11–13^. Therefore, we intended to understand the responses in abandoned pigs to radioactive contamination, which can be helpful in understanding the radiation effects and responses in humans. Our previous report demonstrated that there were alterations in gene expression in the small intestine of animals in the ex-evacuation zone after radiation^4^. The genes involved in inflammation showed significantly higher expression in pigs in the ex-evacuation zone than in control pigs. Therefore, exposed pigs could have an inflammatory response due to oxidative stress with the indirect action of radiation. This is caused by breaking the O–H bonds of water molecules in the body and generating reactive oxygen species^14,15^. As superoxide and hydroxyl radicals of reactive oxygen species have unpaired electrons, they oxidize DNA, proteins, and lipids^16–18^. Consequently, the biomolecules would be damaged. However, the body has a mechanism to eliminate reactive oxygen species. Nevertheless, if numerous reactive oxygen species are generated by radiation, the elimination will be insufficient, leading to oxidative stress. Chronic inflammation due to oxidative stress is known to induce cancer, lifestyle-related diseases, and immune-related diseases. Therefore, we performed a follow-up investigation using wild boars, which are biologically the same species as pigs, in this study. Muscles and small intestines were collected from the wild boars that were exterminated by the Hunting Association. These samples were evaluated for the amount of radioactive cesium, and the changes in the expression of genes responsible for immunological or physiological functions were analyzed (Fig. 1).

## Results

### Radioactivity concentration in skeletal muscles and total exposure dose rates of wild boars

**Figure 2:**
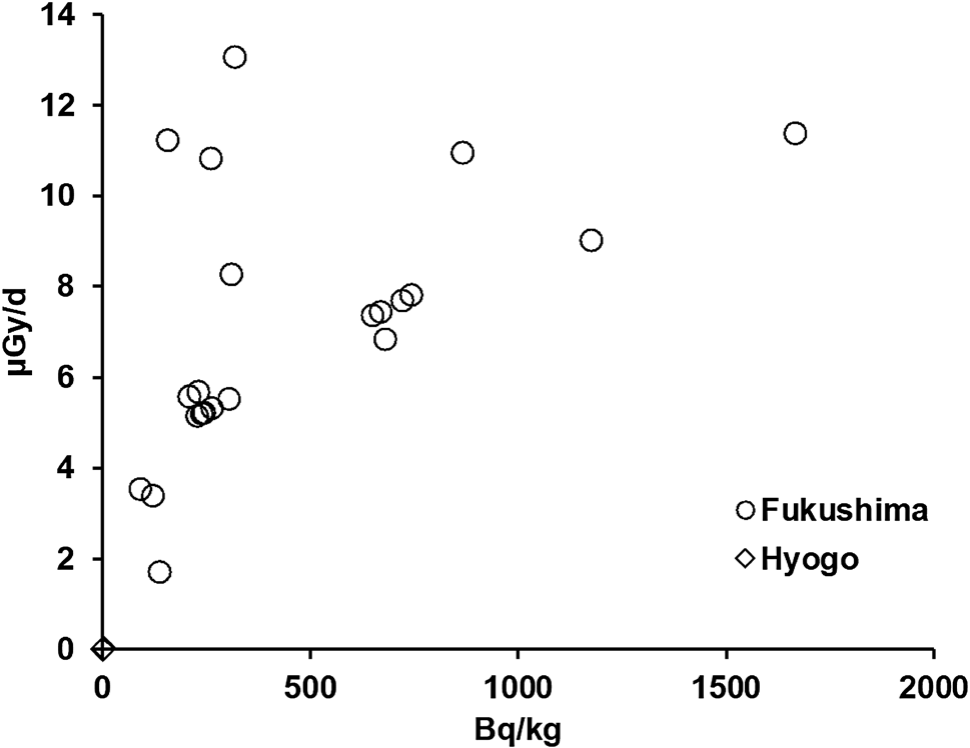
Relation between total exposure dose rate of whole body and ^137^Cs radioactivity concentration in the skeletal muscles of wild boars in Hyogo (*n* = 3) and Fukushima (*n* = 22).

Figure 2 shows relationship between the total exposure dose rates and the radioactivity concentration in the skeletal muscles of wild boars. The total exposure dose rates are summation of internal and external dose rates of whole body. Average ^137^Cs radioactivity concentration and total dose rates in 22 wild boars in the ex-evacuation zone were 470 Bq/kg and 7.2 µGy/d, respectively. The lowest and highest values were 124 and 1667 Bq/kg, respectively. And the medians were 289 Bq/kg and 6.8 µGy/d. In contrast, the average ^137^Cs radioactivity concentration and total dose rates of the three wild boars in Hyogo prefecture were 1.5 Bq/kg and 0.0 µGy. The lowest and highest values were 0.6 and 2.7 Bq/kg, respectively, and the median was 1.2 Bq/kg.

### Gene expressions in the small intestine

**Figure 3:**
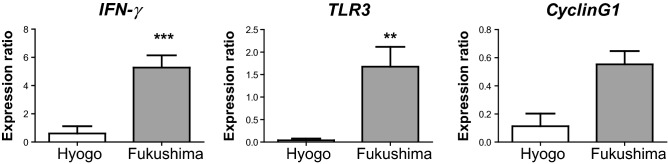
The expression of *IFN-γ*, *TLR3*, and *CyclinG1* in the small intestine of wild boars in Hyogo and Fukushima. The expression ratio of these genes in Fukushima wild boars was significantly higher than that in Hyogo wild boars. All data are expressed in relative units. **P < 0.01, ***P < 0.001, data are presented as mean ± SE.

In our previous study conducted in 2012, microarray analysis revealed that several genes in the small intestine exhibited significant expression differences after radiation in abandoned pigs. More detailed experiments using real-time PCR confirmed that *IFN-γ* and *TLR3* expressions were significantly increased after radiation in abandoned pigs. Furthermore, our subsequent study of wild boars in the ex-evacuation zone in 2015 showed that *CyclinG1* expression was significantly higher than that in the control group^4^. Therefore, we focused on the expression of *IFN-γ*, *TLR3*, and *CyclinG1* in the present study as a follow-up survey. We found that *IFN-γ* and *TLR3* expressions were significantly higher in Fukushima wild boars than in Hyogo wild boars. The expression of *CyclinG1* also tended to be higher (Fig. 3).

### Pathological and morphological changes in the small intestine

**Figure 4:**
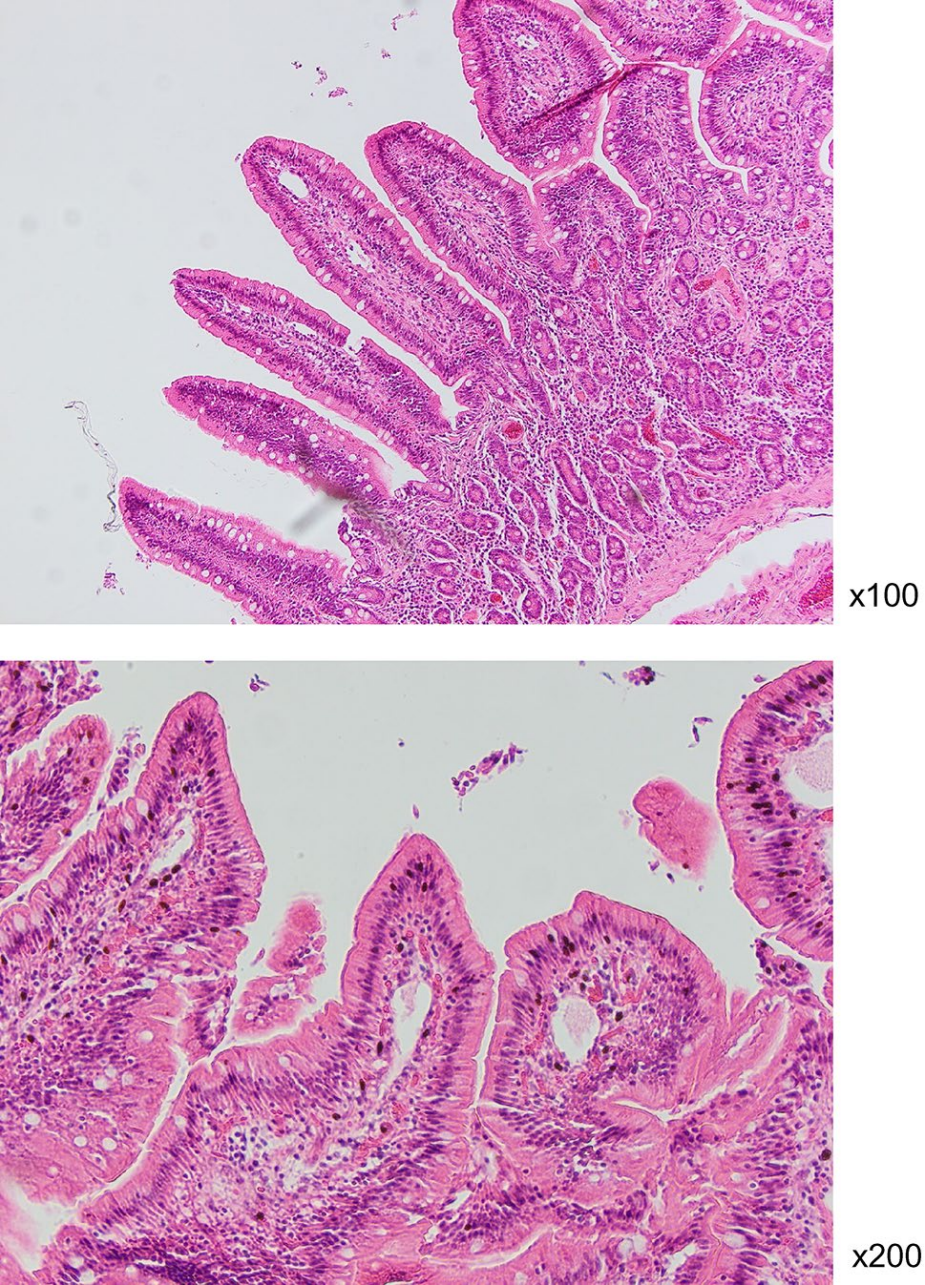
Representative images of hematoxylin and eosin staining of small intestine samples. There were no pathological abnormalities in the small intestine of wild boars in Fukushima.

In the pathological analysis, tissues were fixed and cut for HE staining to examine whether intestinal tissues were damaged or showed inflammation because of radiation exposure. No morphological changes and infiltration of inflammatory cells were observed (Fig. 4).

## Discussion

Although 10 years have elapsed since the earthquake, the reconstruction of the disaster area is in progress. In Fukushima, there are still areas where it is difficult to return home. However, decontamination of urban regions and agricultural land is progressing, and residents are rebuilding their lives. Moreover, agricultural products are sold after being thoroughly inspected for radiation dose and confirmed to be safe. It is the increase in the number of wild animals that threatens the livelihoods of the returning people. From 2016 to 2017, Lyons et al.^8^ surveyed the ecology of wild animals using network cameras. They found that wildlife preferred the environment without humans and increased in number in the ex-evacuation zone, despite chronic radiation exposure. The wild boar was the most abundant species in the ex-evacuation zone. Even before the Fukushima Daiichi accident, wild boars were targeted for extermination, and the Hunting Association was hunting, but at that time, the meat was also edible in this area. However, it is now just discarded after hunting. The wild boars present in the mountains have not been decontaminated but eat contaminated food and water. Several studies on the Chernobyl accident demonstrated that the pollution of mushrooms in the mountain range continued for a long time^19,20^.

The intestine can be significantly affected by radiation through internal exposure after oral intake of contaminated food. It is also one of the essential organs of the immune system. Therefore, we evaluated whether the expression of genes responsible for the immune system and cell cycles in the small intestine of wild boars in the ex-evacuation area is altered compared to that in animals in the noncontaminated area.

Our results demonstrated that *IFN-γ* and *TLR3* were significantly upregulated in Fukushima wild boars compared to those in Hyogo wild boars. Moreover, *CyclinG1* expression tended to increase. As mentioned earlier, these genes were selected from the microarray analysis in our previous research^4^. IFN-γ is one of the crucial cytokines for acquired immunity and inflammation. Recently, Zha et al. described that IFN-γ is a master regulator for several cytokines involved in numerous biological processes^21^. It functions as a master switch to operate cell activation or inhibition. In comparison, the major portion of innate immune cell activation is mediated by TLRs. TLR3 is involved in dsRNA recognition and is associated with antiviral responses. Furthermore, TLR3 is an important molecule for radiation susceptibility. Takemura et al. reported that TLR3-deficient mice exhibited substantial resistance to gastrointestinal syndrome (GIS)^22^. TLR3 is bound to cellular RNA leaking from damaged cells and induces inflammation. CyclinG1 is one of the target genes of the transcription factor p53 and is induced in response to DNA damage. It also plays a role in G2/M arrest in response to DNA damage recovery and growth promotion after cell stress^23^. Therefore, the changes in the expressions of the genes encoding these proteins suggested that the immune system and cell cycles in wild boars in the ex-evacuation zone were affected by low-dose radiation. These results are consistent with our previous investigation conducted in 2012. A state of high *IFN-γ* expression suggests an activated state of immune cells. Despite the low-dose, radiation-induced oxidative stress may result in elevated expression of inflammatory cytokines. However, no correlation was observed between *IFN-γ* expression and radiation levels in the skeletal muscle of wild boars in this study (data not shown). This could be due to the lower doses of ^137^Cs observed in the present study rather than those in the previous investigation. Furthermore, pathological examination revealed no infiltration of immune cells in the submucosa of small intestines of wild boars in the ex-evacuation area.

Therefore, the elevated expression of these genes can be considered as a consequence of the living body’s ability to appropriately process the effects of low-dose radiation. The highest radiation concentration in the skeletal muscle was 1667 Bq/kg, which was much lower than that in abandoned pigs investigated in 2012, at > 15,000 Bq/kg on average. Cui et al. investigated 213 wild boars and reported a median ^137^Cs value of 420 Bq/kg in 2019^10^. Most samples collected from the wild boars in the ex-evacuation zone still showed radioactivity concentration that exceeded the regulatory radiocesium limit for foods in the present study, but the dose is steadily decreasing. Cunningham et al. investigated DNA damage and concluded that there was no evidence of significant harmful impacts to wild boars exposed to low-dose radiation^24^.

Furthermore, Pederson et al. investigated whether chronic low-dose radiation affects cataract prevalence in wild boars but reported no significantly higher risk in the animals in the exclusion zone^25^. Finally, we also report the results of this study as a record of 10 years after the accident. Although an increase in the expression of *IFN-γ*, *TLR3*, and *CyclinG1* was detected, there were no pathological abnormalities in wild boars in the ex-evacuation zone. However, it is difficult to conclude the effects of radiation only ten years after the accident. We intend to continue conducting wild boar surveys regularly to elucidate the effects of long-term low-dose radiation exposure.

## Materials and methods

### Samples

The intestine and muscle samples from 22 wild boars were collected between September 4 and March 2, 2020, in Namie town in Fukushima prefecture. Furthermore, control intestine samples were collected from three wild boars in Hyogo prefecture. Each location is depicted in Fig. 1. In each case, after the licensed hunters slaughtered the wild boar to be exterminated, only the tissue was transferred to the study.

### Measurement of radioactivity

Radioactivity in the muscle samples was determined by gamma-ray spectrometry using high-purity germanium (HPGe) detectors (Ortec Co., Oak Ridge, TN, USA), as described in our previous report^3^. Gamma rays from ^137^Cs were observed.

### Exposure dose estimation

In order to estimate internal and external dose rates of the wild boars according to the ICRP publication 108^26^, we supposed the shapes of wild boars as prolate spheroids whose long axis was to be their body lengths. The short axis was given from their weight assuming their specific gravities were the same as water. The dose rates were calculated from the contribution of 137Cs, not including natural radionuclides. The energy deposition to the spheroids by beta and gamma rays from radionuclides were calculated by the numerical simulation with the use of the Particle and Heavy Ion Transport code System (PHITS)^27^. For the sake of simplicity, we supposed the spheroids consisted of only muscle, which would give overestimated values because muscle contains more radio cesium than other organs. The external exposure dose was calculated from the air dose rates which were observed from the monitoring post near the boars captured place. The average values of the air dose rates were obtained from fitting observed data of two years with decay curve. The background due to the natural radionuclides was estimated to be 0.05 µGy/h which was observed before the Fukushima Daiichi accident, and was removed before the fittings. The half-lives of the air dose rates were 2000–3000 days depending on the environment. Assuming the external exposure dose was ascribed to the ^137^Cs included in the surface of the ground. The amount of the ^137^Cs was calculated so as to reproduce the observed air does rates. Since the maximum range of the beta ray from ^137^Cs is a few millimeters, almost all of the beta ray from inside the body should be absorbed in the boar's body, but the beta ray from outside the body would stop in its fur. The beta rays contribute 100% to internal exposure dose but 0% to external one. Since the linear attenuation coefficient for gamma rays from ^137^Cs is 0.084 cm^−1^ = (12 cm)^−1^, some of the gamma rays cannot stop in the body depending on the size of the body. The numerical simulation suggested that 65–90 percent of the gamma rays from ^137^Cs inside the body would go out, and 40–65 percent of the gamma rays from ^137^Cs outside would go through the body.

### Pathological analysis

A piece of the small intestine was fixed in 10% neutral formalin at 4 °C for 24–48 h. Then, paraffin blocks were prepared for pathomorphological examination using hematoxylin and eosin (HE) staining.

### Gene expression analysis

Total RNA was extracted from the whole tissue of the intestine using TRIzol Reagent (Life Technologies, Inc., Frederic, MD, USA) according to the manufacturer’s instructions. RNA concentration was measured using a NanoDrop spectrophotometer (Thermo Scientific, Wilmington, DE, USA), and cDNA was synthesized using random primers and SuperScript II (Life Technologies, Inc.). Real-time PCR for *IFN-γ*, *TLR3*, and *CyclinG1* was performed using Brilliant SYBR Green QPCR Master Mix III (Stratagene, La Jolla, CA, USA) with an AriaMx system (Agilent Technologies, Santa Clara, CA, USA). Primer sequences were designed using Primer-BLAST with sequences obtained from GenBank as described in the previous report^4^. Amplification conditions were 95 °C for 3 min, 40 cycles at 95 °C for 5 s, and 60 °C for 20 s. Fluorescence signals measured during the amplification were analyzed. Ribosomal RNA primers were used as an internal control, and all data were normalized to constitutive rRNA values. Quantitative differences between the groups were analyzed using the AriaMx software (Agilent Technologies).

### Statistical analysis

All data are presented as mean ± standard error (SE) for each treatment group. Differences in mRNA expression among the groups were determined using the unpaired *t*-test with Welch's correction. (Prism: GraphPad Software Inc., La Jolla, CA, USA). Differences were considered to be statistically significant at a *P* value of < 0.05.

### Ethics approval

No animals were killed for this research. Use of all animals was secondary to control wildlife pests. We conducted the investigation with the permission of the local government.

## Data Availability

The datasets generated and analyzed during the current study are available from the corresponding author upon reasonable request.
